# Development of a five-plex flow cytometric immunoassay for the simultaneous detection of six coccidiostats in feed and eggs

**DOI:** 10.1007/s00216-012-6214-1

**Published:** 2012-08-01

**Authors:** Monique E. Bienenmann-Ploum, Anne-Catherine Huet, Katrina Campbell, Terence L. Fodey, Ursula Vincent, Willem Haasnoot, Philippe Delahaut, Christopher T. Elliott, Michel W. F. Nielen

**Affiliations:** 1 RIKILT-Institute of Food Safety, Wageningen UR, P.O. Box 230, Wageningen, 6700 AE The Netherlands; 2 Centre d’Economie Rurale-CER Groupe, Département Santé, Rue du point du jour, 8, 6900 Marloie, Belgium; 3 Institute of Agri-Food and Land Use, School of Biological Sciences, Queen’s University Belfast, David Keir Building, Stranmillis Road, Belfast, BT9 5AD UK; 4 European Commission, Joint Research Centre, Institute for Reference Materials and Measurements (EC-JRC-IRMM), 2440 Geel, Belgium; 5 Laboratory of Organic Chemistry, Wageningen University, Dreijenplein 8, Wageningen, 6703 HB The Netherlands

**Keywords:** Coccidiostats, Multiplex flow cytometric immunoassay, Colour-coded beads, Eggs, Feed

## Abstract

Coccidiostats are the only veterinary drugs still permitted to be used as feed additives to treat poultry for coccidiosis. To protect consumers, maximum levels for their presence in food and feed have been set by the European Union (EU). To monitor these coccidiostats, a rapid and inexpensive screening method would be a useful tool. The development of such a screening method, using a flow cytometry-based immunoassay, is described. The assay uses five sets of colour-coded paramagnetic microspheres for the detection of six selected priority coccidiostats. Different coccidiostats, with and without carrier proteins, were covalently coupled onto different bead sets and tested in combination with polyclonal antisera and with a fluorescent-labelled secondary antibody. The five optimal combinations were selected for this multiplex and a simple-to-use sample extraction method was applied for screening blank and spiked eggs and feed samples. A very good correlation (*r* ranging from 0.995 to 0.999) was obtained with the responses obtained in two different flow cytometers (Luminex 100 and FLEXMAP 3D). The sensitivities obtained were in accordance with the levels set by the EU as the measured limits of detection for narasin/salinomycin, lasalocid, diclazuril, nicarbazin (4,4′-dinitrocarbanilide) and monensin in eggs were 0.01, 0.1, 0.5, 53 and 0.1 μg/kg and in feed 0.1, 0.2, 0.3, 9 and 1.5 μg/kg, respectively.

**Figure:**
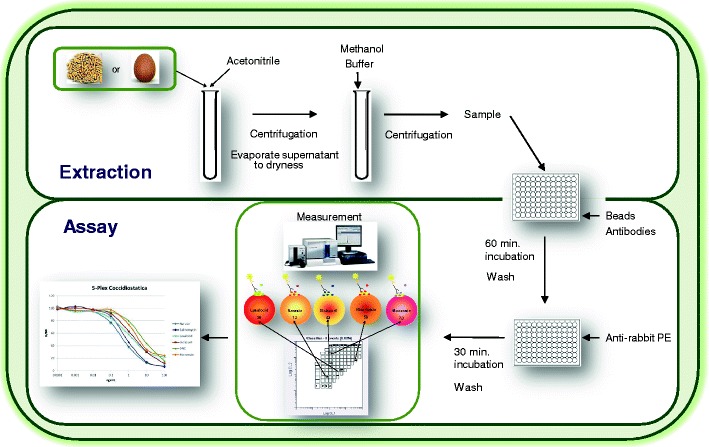

## Introduction

Coccidiosis is a protozoan infection of the intestinal tract, caused by protozoa belonging to the genus *Eimeria*, which especially affects poultry. These infections result in economic losses because of decreased growth rate, reduced egg production and increased mortality [1]. To control this disease, a range of different coccidiostats are used as feed additives. Currently, 11 different coccidiostats are authorised in the European Union (EU) for the prevention of coccidiosis in chickens, turkeys and rabbits (Regulation (EC) No 1831/2003) [2]. These coccidiostats can be grouped into two major types. The first group includes the polyether group-containing ionophores which are produced by micro-organisms and comprises the six following substances: monensin, lasalocid, maduramicin, narasin, salinomycin and semduramicin. Their biological activity is based on their ability to form lipid-soluble and dynamically reversible complexes with cations. The second group includes the synthetic products not of an ionophoric nature: decoquinate, robenidine, halofuginone, diclazuril and nicarbazin (equimolar complex of 4,4′-dinitrocarbanilide (DNC) and 2-hydro-4,6-dimethylpyrimidine (HDP)). DNC is the anticoccidial active substance, but this activity increases when DNC is administered as a complex with HDP. Moreover, the excretion of DNC by chickens is slower than for HDP and DNC was therefore selected as a marker for residue detection [3].

These coccidiostats are not allowed to be used in egg-laying chickens and in broilers 5 days before slaughter. Maximum (residue) levels for the presence of coccidiostats in food (maximum residue limits (MRLs); Commission Regulation (EC) 124/2009 [4] and Commission Regulation (EC) 1353/2007 [5]) and feed (maximum limits (MLs); Commission Regulation No. 574/2011) [6] have been set within the EU (Table 1), to protect animals and animal products from the unintentional treatment induced by carry-over rates of feed produced with the highest authorised dose of the coccidiostats into the afterwards produced non-target feed. The 11 authorised coccidiostats are included into the National Monitoring Programme (96/23/EC) [7] and EU member states are obliged to monitor eggs and feed samples for their presence.

**Table 1:** Maximum (residue) levels for coccidiostats in eggs (MRLs) and feed (MLs; EU legislation) [4–6]

	Eggs (μg/kg)	Feed (μg/kg)
Lasalocid sodium	150	1,250
Monensin sodium	2^a^	1,250
Salinomycin sodium	3	700
Narasin	2	700
Nicarbazin	100	500/1,250^b^
Diclazuril	2	10
Semduramicin sodium	2^a^	250
Maduramicin	2^a^	50
Robenidine	25	700
Decoquinate	20^a^	400
Halofuginone	6	30

^a^Not specified for eggs but as ML in other food of animal origin

^b^New ML since 2011

To monitor these coccidiostats, two different types of methodologies have been developed to detect one or more coccidiostats simultaneously. Firstly chromatography-based methods such as high-performance liquid chromatography [8, 9] and liquid chromatography-mass spectrometry (LC-MS) [1, 10–15] and secondly immunoassays [10, 16–21] including lateral flow devices [22] and a surface plasmon resonance-based biosensor [23]. During the last number of years, chromatography-based methods have improved with respect to reduced analysis time (including sample preparation time) and multiplexed detection. However, immunoassays still have several advantages over physico-chemical techniques such as their lower cost, their ease-of-use and speed. Yet to date virtually all of these immunoassays can only detect one or two of the coccidiostats simultaneously.

The flow cytometry-based immunoassay (FCIA) applied in the present study is an interesting suspension array format, combining the favourable properties of an immunoassay with the opportunity of combining assays (multiplexing). This FCIA uses paramagnetic microspheres, which are internally dyed with a red and an infrared fluorophore. By varying the ratio of the two fluorophores, up to 100 different colour-coded bead sets can be distinguished and different compounds or conjugates can be coupled on the surfaces of different bead sets.

The carboxylated bead surface allows simple chemical coupling of capture reagents such as antibodies, oligonucleotides, proteins, peptides, polysaccharides or receptors. For the coating of the beads with proteins, standard procedures are available [24]. The coating with low molecular weight compounds is usually accomplished by conjugating them to a carrier protein. Such procedures have been described for the detection of pesticides [25], the thyroid hormone thyroxine [26], veterinary drug residues [27, 28], polycyclic aromatic hydrocarbons [29, 30], mycotoxins [31] and explosives (TNT) [32]. The direct coupling of a low molecular weight sulfonamide to the beads was also described by de Keizer et al [33], and this resulted in an improved bead stability with higher responses and an easier coupling procedure.

Antibodies can bind to these immobilised compounds or conjugates on the beads and these binding events will be inhibited by the presence of free compounds in the sample in the applied inhibition immunoassay format. The bound antibodies are quantified by a secondary antibody (IgG) labelled with the fluorescent protein R-phycoerythrin (PE). Inside the dual-laser flow cytometer, one light source (red laser) excites the internal dyes that identifies each microsphere, and one (green laser) quantifies the PE reporter dye on the surface of the bead reflected as median fluorescence intensity (MFI). This makes it possible to simultaneously measure up to 100 different compounds in a single well.

**Fig. 1:**
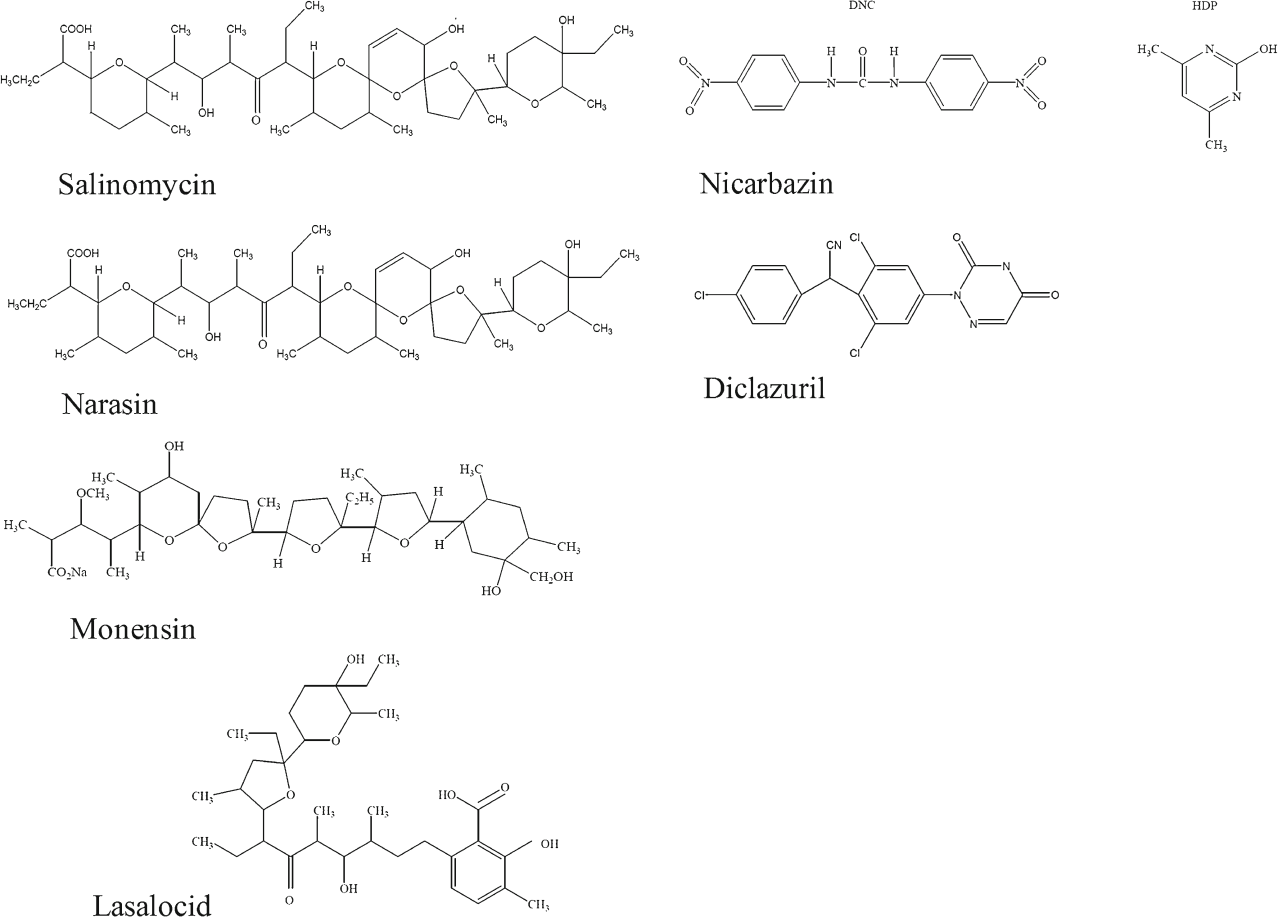
Molecular structures of the six priority coccidiostats

In the present study, a very attractive high throughput multiplex FCIA was developed for the simultaneous detection of six priority coccidiostats (Fig. 1) in eggs and feed. Currently, five bead sets are used for this assay but new developed assays can be added at any time to broaden the scope of this multiplex. The multiplex includes the four polyether ionophores, salinomycin, monensin, narasin and lasalocid, which are most commonly used in poultry [34],[35]. The fifth compound is nicarbazin, a synthetic complex composed of an equimolar amount of DNC and HDP. The sixth compound included within the assay is diclazuril, which belongs to the group of triazinones and is active against intracellular development stages of coccidia.

During the development of the five-plex immunoassay, the coccidiostats with and without carrier proteins, were covalently coupled onto different types of carboxylated beads (paramagnetic and non-paramagnetic beads) and compared for their performances in two different flow cytometers and in combination with new and previously developed polyclonal antisera. The five optimal combinations were selected for the multiplex development. Cross-interactions between the assays and cross-reactivities within the assays were examined. For the assay which detects both narasin and salinomycin, special attention was paid to select a combination with an equal sensitivity for both compounds. Different assay parameters such as, concentrations of the reagents, incubation temperatures and time and buffer composition were trialled. Following this optimisation phase, blank and samples spiked egg and feed samples at relevant concentrations, incurred egg and contaminated feed samples were used to optimise the simplified extraction method and to confirm that levels set by the EU could be detected.

## Materials and methods

### Materials

The HBS-EP buffer and an amine coupling kit (containing 0.1 M *N*-hydroxysuccinimide (NHS) and 0.4 M 1-ethyl-3-(3-dimethylaminopropyl) carbodiimide hydrochloride (EDC)) were supplied by GE Healthcare (Uppsala, Sweden). Ethylenediamine (EDA), dimethyl sulfoxide (DMSO), dimethylformamide (DMF), acetonitrile, methanol, l-glutamic acid gamma-(*p*-nitroanilide) (GAN), sodium carbonate (Na_2_CO_3_), sodium hydrogen carbonate (NaHCO_3_), sodium dihydrogen phosphate monohydrate, Tween-20 and sodium azide were obtained from VWR International (Amsterdam, The Netherlands). Goat-anti-rabbit-R-phycoerythrin conjugate (GAR-PE) was from Molecular Probes (Leiden, The Netherlands) and bovine serum albumin (BSA), bovine thyroglobulin (BTG) and ovalbumin (OVA) from Sigma-Aldrich (Zwijndrecht, The Netherlands). EDC, 4-(2-hydroxyethyl)-1-piperazineethanesulfonic acid and *N*-hydroxysulfosuccinimide sodium salt (sulfo-NHS) were from Pierce (Rockford, IL, USA). The carboxylated microspheres No. 012, 021, 029, 043, 059 and 070 (SeroMAP™ (non-paramagnetic) and MagPlex® (paramagnetic) beads), in stock bead suspensions of 1.25 × 10^7^ beads mL^−1^ and stored at 4–6 °C, and sheath fluid were obtained from Luminex (Austin, Texas, USA). *N*-succinyl-l-alanyl-l-alanyl-l-alanine 4-nitroanilide (SAN) was from Fluka (Gillingham, Dorset, UK). Protein A Sepharose 4 Fast Flow from GE Healthcare (Sweden) was used for the purification of the sera.

Blank and incurred eggs and poultry feed samples were supplied by CER (Marloie, Belgium), FERA (York, UK) and the Nutreco company MasterLab B.V. (Boxmeer, The Netherlands).

### Instrumentation

The measurements were performed using two flow cytometry platforms. The Luminex® 100 IS 2.2 system, supplied by Applied Cytometry Systems (Dinnington, Sheffield, South Yorkshire, UK), consisted of a Luminex 100 analyser and a Luminex XY Platform and operated with StarStation System control software. The FLEXMAP 3D® system, controlled with xPONENT 4.0 Software Solutions, was obtained from Luminex (Austin, Texas, USA).

All washing steps were carried out on a Bio-Plex™ Pro II Wash Station (Bio-Rad Laboratories, Veenendaal, The Netherlands) using a magnetic plate carrier or the vacuum carrier. Plates were incubated on a Dynatech microtiter vari-shaker (Alexandria, VI, USA). During the coupling procedures, the paramagnetic microspheres were captured using a DynaMag-2™ (Invitrogen Dynal, Oslo, Norway) magnetic separator. Mixing for sample extraction was performed in a REAX2 (Heidolph, Schwabach, Germany) overhead shaker. All centrifuge steps were carried out in an Eppendorf 5810 R centrifuge using the A-4-62 rotor (VWR International, Amsterdam, The Netherlands).

### Methods

#### Preparation of immunoreagents and antisera

Polyclonal antisera were obtained from rabbits after immunisations with the different coccidiostats conjugated to the carrier proteins BTG, BSA or transferrin in which three different conjugation chemistries were applied. These chemistries were performed at the Institute of Agri-Food and Land Use of the Queen’s University Belfast (Belfast, UK) and were also applied for the couplings to OVA or horse-radish peroxidase (HRP).

*The N,N′-disuccinimidyl carbonate (DSC) reaction* was applied for the conjugation of diclazuril, lasalocid and salinomycin to proteins. DSC consists of a carbonyl group containing two NHS esters. The compound is highly reactive towards nucleophiles and, in non-aqueous conditions, it can be used to activate a hydroxyl group to a succinimidyl carbonate derivative. DSC-activated hydroxyl compounds can then react with amine-containing molecules to form stable cross-linked products. For the activation, the drug was mixed with a six times molar excesses of DSC and dimethylaminopyridine, all dissolved in dry acetone. After mixing for 3–4 h at room temperature (RT), the acetone was evaporated to dryness under a stream of nitrogen and the activated drug was re-suspended in PBS (pH 7.5). DMSO or pyridine was used if the product was insoluble in water. The activated drug was added in a molar ratio equivalent to the number of amines groups to the protein (50 mg) dissolved in PBS and incubated for at least 4 h at RT or overnight (ON) at 4 °C.

*The N,N′-carbonyldiimidazole (CBDI) reaction* was applied for the conjugation of lasalocid, monensin, salinomycin, narasin and GAN (a mimic of the active component DNC in nicarbazin) to proteins. The activation of the carboxylate group with CBDI provides an intermediate imide with imidazole as the active leaving group. In the presence of a primary amine-containing compound, the nucleophile attacks the electron-deficient carbonyl, displacing the imidazole and forming a stable amide bond. The drug dissolved in DMF was added to a six times molar excess of CBDI dissolved in acetone and stirred at RT for 4 h and the acetone was then evaporated. The protein (10–20 mg/mL) was dissolved in carbonate/bicarbonate buffer (pH 9.6), and the activated drug was slowly added whilst stirring. The mixture was incubated ON at 4 °C.

*The carbodiimide reaction* was applied for the conjugation of diclazuril and SAN (another mimic of the active component DNC in nicarbazin). In this, EDC reacts with carboxylic acids (hapten or protein) to form highly active *O*-acetylisourea intermediates which can then react with a primary amine to form an amide bond and release EDC as a soluble isourea derivative. NHS can be used with EDC to increase the stability of the active intermediate. The drug (10 mg) was dissolved in 0.05 M 2-(*N*-morpholino)ethanesulfonic acid (MES) and 0.5 M sodium chloride (pH 4.7) or pyridine. EDC (10 mg) and NHS (5 mg) dissolved in 0.005 M MES; 0.5 M sodium chloride (pH 4.7) were added to the drug and mixed for 15 min. The protein (20 mg) was dissolved in PBS and added to the activated drug dropwise and allowed to incubate ON at RT.

All the protein-drug conjugates were purified by overnight dialysis against 0.15 M saline over 24 h (3 × 4 L).

*Immunisations* were performed at CER (Marloie, Belgium) and at least five rabbits were injected with the same immunogen which was received in lyophilised form and was reconstituted to 1 mg/mL with water for injection. For each injection, 0.2 mg of the immunogen was emulsified with NaCl (0.9 %) and Freund’s adjuvant by mixing vigorously. A complete adjuvant was used only for the first immunisation and incomplete adjuvant for all subsequent booster injections. The emulsified antigens were injected subcutaneously at four sites on the New Zealand white-specific pathogen-free rabbits at days 0, 14 and 28 and then every 28 days, with test bleeds taken from the marginal ear vein 10 days after immunisations. These bleeds were collected from the third immunisation onwards. The blood was centrifuged and collected serum was stored at −20 °C until further use.

#### ELISA for evaluation of sera

Two methods were used for the evaluation of the presence of antibodies in the sera of the immunised rabbits.

In the first assay format, the microtitre plate was coated with purified sheep anti-rabbit IgG and diluted sera as well as HRP-drug conjugate were added and incubated ON at 4 °C. After washing, colour developed was achieved by given a 30-min incubation of TMB/H_2_O_2_. The reaction was stopped by the addition of H_2_SO_4_, and the colour intensity was measured at 450 nm.

In the second assay format, the diluted sera were coated to the microtitre plate and buffer or standard solution as well as the HRP conjugate were added and incubated for 2 h at 37 °C. After washing, colour development was performed as described for assay format 1.

##### Immobilisation of coccidiostat-protein conjugates on the beads

HRP, BSA or OVA conjugates of lasalocid, monensin, salinomycin, narasin, diclazuril and GAN were coupled to MagPlex® paramagnetic carboxylated beads, and each to a unique bead set, according to a standard Luminex protocol for protein coupling [24]. The concentrated bead suspension (1.25 × 10^7^ beads/mL) was vortexed vigorously for 5 min and then placed in an ultrasonic bath for 1 min. For each bead coupling, 200 μL of the concentrated beads was transferred to an Eppendorf tube and placed in the magnetic separator. Beads were allowed to settle for 3 min and the supernatant was aspirated. The beads were washed by 500 μL MQ water added, mixed for 1 min by vortex, placed in an ultrasonic bath for 1 min and in the magnetic separator for 3 min and aspirating the supernatant. This was the standard washing procedure for the paramagnetic beads. The beads were subsequently washed with 500 μL of 0.1 M NaH_2_PO_4_ (pH 6.3), activated for conjugate coupling in 100 μL of 0.1 M NaH_2_PO_4_ (pH 6.3) containing 500 μg of EDC and 500 μg of NHS and incubated for 20 min at room temperature. After activation, the beads were captured and washed once with 500 μL PBS (5.4 mM sodium phosphate, 1.3 mM potassium phosphate and 150 mM sodium chloride; pH 7.4). Final conjugate concentrations were adjusted to 125 μg/mL in PBS. The activated beads were then resuspended in 0.5 mL of the conjugate solution. Subsequently, they were incubated for 2 h at RT in the dark with gentle agitation. After the coupling, the conjugate solutions were removed and the beads were washed four times with blocking buffer (PBS buffer containing 0.1 % BSA, 0.02 % tween 20 and 0.02 % of sodium azide). The beads were then resuspended in 200 μL blocking buffer and stored ON at 4 °C before use.

##### Direct immobilisation of coccidiostats or mimics on the beads

Lasalocid, monensin, salinomycin and GAN were directly coupled to paramagnetic (MagPlex®) or non-paramagnetic beads (SeroMAP™), and each to a unique bead set, using a previously described procedure [33] in which the carboxyl groups were activated by EDC/NHS. For each coupling, 0.5 g of coccidiostat was dissolved in 160 μL DMSO or DMF and the volume adjusted to 800 μL with carbonate buffer (15 mM Na_2_CO_3_ and 35 mM NaHCO_3_ at pH 9.6). To 150 μL of this solution, 150 μL EDC and 150 μL NHS (amine coupling kit) were added and this mixture was incubated for 1 h in the dark before it was added to the activated beads. The concentrated bead suspensions were pretreated as described previously and 200 μL was transferred to an Eppendorf tube and placed in a magnetic separator (paramagnetic beads) or centrifuged for 3 min at 10,000×*g* for the non-paramagnetic beads. The paramagnetic beads were washed as described above. For the non-paramagnetic beads 500 μL MQ water were added, the mixture was vortexed for 1 min, placed in an ultrasonic bath for 1 min and finally centrifuged for 3 min at 10,000×*g*. This was the standard washing procedure for the non-paramagnetic beads. The beads were subsequently washed and activated as described previously. The activated beads were captured magnetically or centrifuged and the supernatant removed. The pellet was resuspended in 250 μL of 0.1 M EDA (pH 8.5). After 15 min shaking at RT in the dark, the beads were captured or centrifuged and the supernatant was discarded. The pellet was washed twice with 500 μL PBS. Finally, the beads were re-suspended in the 450 μL of activated coccidiostat solution. The mixture was incubated for 2 h in the dark in a test tube rotator, centrifuged and the supernatant was removed. After the coupling, the drug solutions were removed and the beads were washed and resuspended in blocking buffer as described above.

#### Extraction procedure for feed and eggs

Homogenised whole eggs or grinded feed (2 g) were mixed with 8 mL of acetonitrile by immediately vortexing for 30 s. The sample was vigorously shaken on an overhead shaker for 10 min and centrifuged at 2,400×*g* for 10 min. Of the supernatant, 4 mL were transferred to another tube and evaporated to dryness under a flow of nitrogen at 50 °C. After the addition of 0.2 mL of methanol, the sample was vortexed for 10 s. Subsequently, 0.8 mL of HBS-EP buffer was added, the sample was vortexed for 10 s and vigorously shaken for 10 min, followed by a centrifugation at 2,400×*g* for 10 min.

#### Flow cytometric immunoassay protocol

##### Paramagnetic beads

Calibration curves of the individual coccidiostats (narasin, salinomycin, lasalocid, diclazuril, nicarbazin (DNC) and monensin) or of a mixture of coccidiostats were prepared in HBS-EP buffer containing 10 % methanol. Bead suspensions, with concentrations of around 100 beads μL^−1^ bead set^−1^, and antibody dilutions (individual or as mixture) were made in 0.1 % BSA blocking buffer [24]. Subsequently, 10 μL of this bead suspension, 50 μL sample extract, plus 50 μL HBS-EP buffer containing 10 % methanol (or 50 μL blank sample extract plus 50 μL calibration standard solution) and 10 μL of antibody solution were added to a well of the flat-bottom 96-well plate and was incubated on the microplate shaker in the dark for 1 h. After washing three times with PBS in the Bio-Plex washer, 70 μL of 600 times diluted GAR-PE was added followed by an incubation on the microplate shaker in the dark for 30 min. The beads were analysed with a Luminex analyser in which about 100 beads per bead set were counted. The antibody-binding was quantified by the response (MFI) obtained from the amount of reporter molecule (PE) and the amount of cocciostats in the samples were quantified by the individual calibration curves prepared using GraphPad Prism software, paramagnetic beads

##### Non-paramagnetic beads

A very similar protocol to that applied for the paramagnetic beads was used, except that 96-well filter-bottom plates were utilised which were pre-wetted with 100 μL of 0.1 % BSA in HBS-EP and aspirated before use. A second difference was the washing step where the plate was only washed twice, using the vacuum carrier, and finally, 100 μL of 1/600 diluted GAR-PE was added.

## Results and discussion

### Antisera development

For each of the five different coccidiostat assays, antisera from between 5 to 15 rabbits were available for testing and for most assays with different coated beads.

Salinomycin and narasin have almost identical chemical structures (Fig. 1) as well as identical MRLs in eggs and MLs feed (Table 1). The aim was therefore to develop an assay with similar sensitivity for both coccidiostats. Of all the antisera from the rabbits immunised with salinomycin-BTG (*n* = 5) and narasin-BTG (*n* = 5), none showed colour development in the ELISA. FCIAs could be made with four of these antisera when beads were coated with their respective conjugates. However, low cross-reactivities were observed with the two structures. The best combination was anti-salinomycin (CF6) combined with narasin-BSA-coated beads, which resulted in a reasonable response (4,000 MFI) and antibody dilution (1:400), showing a 150 % cross-reactivity for narasin compared with salinomycin with a high sensitivity (IC_50_ for narasin and salinomycin at 0.3 and 0.5 ng/mL, respectively).

For the production of polyclonal sera against monensin, two attempts with each five rabbits immunised with monensin-BTG (CF1-CF5) or monensin-CBDI-BTG (CF141-CF145), and one attempt with five rabbits immunised with monensin-CBDI-BSA (CF146-CF150) were done. They did not present any satisfying results in the applied ELISAs. Likewise no (*n* = 6) or low (*n* = 9) responses were seen with the FCIA using the direct monensin-coated beads. When monensin-OVA-coated beads were used, high responses were observed but with a very low sensitivity (IC_50_ for monensin, >3,000 ng/mL). A new monensin-BSA conjugate was therefore produced, coupled to the beads and performed best with the antisera CF141-CF145 (monensin-CBDI-BTG). For further optimisation one of the antisera (CF141) was affinity purified using a protein A column. These modifications had a positive effect on the sensitivity of the assay (IC_50_ of 0.3 ng/mL) and the maximum response obtained (4,000 MFI).

With three of the antisera of the rabbits (*n* = 5) immunised with diclazuril-BTG, binding was observed in the ELISA and FCIA however, the IC_50_ values were high (100–900 ng/mL for the ELISA and >3,000 ng/mL for the FCIA). A previously developed antiserum against carboxy-diclazuril-BTG (CT28) gave a better sensitivity in the ELISA (IC_50_ of 5.2 ng/mL for free diclazuril). Similar results were seen in the FCIA and the best combination was CT28 with diclazuril-HRP coated beads (IC_50_ of 1.9 ng/mL).

Of the rabbits immunised with lasalocid-protein conjugates (*n* = 15), only a few showed low binding in the ELISA and the FCIA of which one (CF131) showed a good sensitivity (IC_50_ of 0.5 ng/mL) in combination with lasalocid-OVA-coated beads.

The rabbits (CF21–CF25) immunised with SAN-BTG showed binding in the ELISA with reasonable sensitivities (IC_50_ values for DNC of 20 to 1,000 ng/mL) and two of them (CF21 and CF22) performed well in the Luminex with the structural mimic GAN-OVA-coated beads (IC_50_ of 4–9 ng/mL).

### Singleplex assay development

The goal of this research was to develop a multiplex FCIA for the simultaneous detection of six different coccidiostats at or below the MRLs and MLs prescribed within the EU for eggs and feed (Table 1). Different protein conjugates were made for the immunisations (BTG or BSA) of the rabbits and for the coupling to the beads (HRP or OVA). Of the three available bead types, all detectable in the two applied flow cytometers (Luminex® 100 and the FLEXMAP 3D®), the MicroPlex® beads were not used because of the possible problems with non-specific binding [36]. The SeroMAP™ beads (non-paramagnetic beads) were designed for reducing these non-specific interactions, especially in serological assays. The MagPlex® beads are paramagnetic with the advantage that they are easier to handle during couplings but also during analysis in which the beads can be trapped on the bottom of the well by a magnet during the washing procedure. This gives the opportunity to remove unwanted sample compounds from the well. The non-paramagnetic beads have to be used in combination with filter plates during the washing procedures and unwanted sample compounds will stay in the well. The other differences between the two chosen bead types were the diameters (6.5 μm for the paramagnetic beads versus 5.6 μm for the non-paramagnetic beads) and the surfaces of which the paramagnetic beads are more irregular leading to a larger surface area, an expected larger number of carboxyl groups on the surface and higher coupling quantities. The bead-coating with low molecular weight compounds is usually achieved via carrier proteins. However, as shown before [33], a direct coupling of a low molecular weight compound to the bead could improve the stability of the coupled beads. Therefore, three coccidiostats and one mimic (GAN), all containing a carboxyl group, were directly coupled to the two bead types with EDA as the spacer and their performances in the individual FCIAs, with a selection of antisera, were compared with those applying paramagnetic beads coated with protein conjugates (Table 2). Diclazuril was not included in this comparison due to the more complex coupling procedure. However, the direct coupling onto paramagnetic beads showed insufficient responses in all four assays, and could not be used for further assay development. With the beads, the direct coupling resulted in only two suitable assays (Table 2). Overall, the assays based on protein-conjugate-coupled paramagnetic beads showed higher responses at higher antisera dilutions resulting in higher sensitivities and lower IC_50_ values (Table 2). The used conjugates had substantial influences on the sensitivities and the maximum signals of the assays. This might also explain the difference found between the results obtained with the FCIAs and ELISAs.

**Table 2:** Comparison of sensitivities (IC_50_ value), antibody dilutions and maximum responses (MFI), in the Luminex 100 flow cytometer, of the singleplex assays using two different beads couplings and the optimal dilutions of the polyclonal antisera

Coccidiostat on the bead	Antibody	Direct coupling (non-paramagnetic beads)			Protein conjugate coupling (paramagnetic beads)		
		IC_50_ (ng/mL)	Antibody dilutions	Max MFI	IC_50_ (ng/mL)	Antibody dilutions	Max MFI
Salinomycin	CF6	>5,000	80	4,500	0.3	1,200	4,000
GAN (nicarbazin mimic)	CF21	4	500	5,000	9	1,000	20,000
Monensin	CF141	25	200	4,000	0.3	1,000	4,000
Lasalocid	CF131	–	100	300	0.5	200	2,000

### Five-plex immunoassay specificity and sensitivity of the assays

The five singleplex assays had to be combined within a single well to create the multiplex format. Therefore, a high specificity of the antibodies for their corresponding beads was mandatory and cross-interactions between the assays had to be avoided as much as possible. During the first experiments, such cross-interactions were observed from the monensin and lasalocid antibodies to the narasin and nicarbazin/DNC beads. This problem was solved by using paramagnetic beads for all assays, narasin- and monensin-BSA conjugates and protein A-purified monensin and lasalocid antibodies. An unexpected advantage of this purification was the increased assay sensitivity by a factor of 10 and 2, respectively. After these changes, the mixture of the five bead sets was again incubated with the selected individual antisera (Table 3) to check for cross-interactions.

**Table 3:** The optimal combination of conjugated beads and antibodies

Coccidiostat	Protein conjugate on the beads	Bead conjugate chemistry	Antibody used	Immunogen	Immunogen conjugate chemistry
Salinomycin	Narasin-BSA	CBDI	CF6	Salinomycin-BTG	DSC
Narasin	Narasin-BSA	CBDI	CF6	Salinomycin-BTG	DSC
Diclazuril	Diclazuril-HRP	DSC	CT28	Carboxy diclazuril	Carbodiimide
Monensin	Monensin-BSA	CBDI	CF141	Monensin-BTG	CBDI
Lasalocid	Lasalocid-OVA	DSC	CF131	Lasalocid-BTG	CBDI
Nicarbazin	GAN-OVA	CBDI	CF21	Nicarbazin (SAN)-BTG	Carbodiimide

**Fig. 2:**
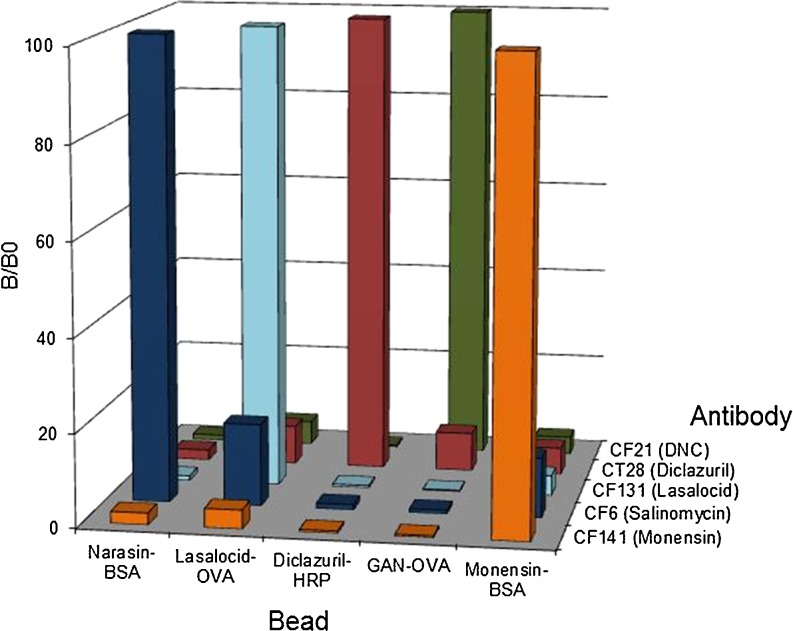
Binding of the five individual polyclonal antisera to the five different beads of the five-plex

**Fig. 3:**
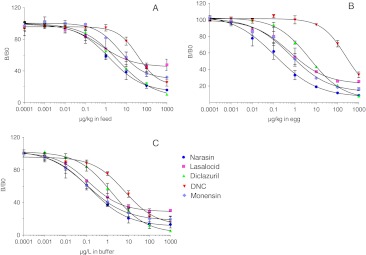
Average calibration curves of the coccidiostats in the five-plex (using mixed calibration standards of the five coccidiostats) in feed (**A**) and eggs (**B**), measured on 2 days, and buffer (**C**), measured on 3 days

As shown in Fig. 2, the antibodies reacted mainly to their corresponding beads. The diclazuril antiserum showed low cross-interaction with all other beads (<10 %). The salinomycin antiserum had a low binding with the lasalocid beads and less with the monensin beads. The monensin antibody now showed low cross-interactions with all other beads due to the purification and high dilution of the antiserum. The diclazuril bead showed the lowest interactions with the other antibodies due to the high maximum MFI (20,000 MFI) obtained with the anti-diclazuril. Although these interactions were all below 20 %, together they had an influence on the calibration curves as these interactions could not be inhibited by any of the coccidiostats. As shown in Fig. 3C, the highest inhibition was obtained with the diclazuril assay of which the beads showed no interactions with other antisera. The lowest inhibition was obtained with the lasalocid assay of which the beads showed the most interactions with the other antisera. The antibody-binding was quantified by the reporter molecule (PE) and expressed in MFI which was corrected for intra-assay and daily inter-plate fluctuations by calculating the percentage of relative binding (*B*/*B*_0_) calculated from the maximum response (*B*_0_) obtained without any analyte.

To test for cross-reactivities towards the 11 authorised coccidiostats (Table 1), a high concentration (1,000 ng/mL) of each coccidiostat in buffer was analysed in the five-plex and the only significant inhibition seen (>30 %) was for narasin in the salinomycin assay. The chemical structures of narasin and salinomycin only differ by one methyl group and the high cross-reactivity (150 % for narasin), obtained by comparing dose–response curves, was expected. The chemical similarities of the other nine authorised coccidiostats are low and therefore cross-reactivities were, as expected, low (<1 %).

Nicarbazin is a synthetic complex of an equimolar amount of DNC and HDP. The excretion of DNC by chicken is slower than for HDP and DNC was selected as a marker compound for residue detection. DNC will be present in eggs where nicarbazin has been added to laying hens feed. This assay had to be applicable to both eggs and feed analysis and proved to be fit for purpose in both matrices to reliably detect DNC reflecting the initial presence of nicarbazin at or below the levels set in legislation.

### Assay performance in sample materials

Ideally, the five-plex screening method should have a similar extraction procedure for both egg and feed samples. Different extraction procedures, described for MS analysis [13, 14], were selected, simplified by performing only the liquid–liquid extraction and tested. Different assay circumstances were tested and a reduction of the first incubation step was not an option because this resulted in higher standard deviations. Temperature adjustments had only a small impact and RT was selected to minimise the necessary equipment. Different buffers, like PBS and HBS could be used without any problem if a detergent or a 0.1 % BSA or OVA was added. The optimal assay and extraction procedures (see “Materials and methods”) were selected for sensitivity, recovery, usefulness for eggs and feed, costs, easy to use and time consumption.

Matrix influences were investigated with calibration curves of the five mixed coccidiostats in buffer, egg and feed extracts (Fig. 3). Substantial differences in assay performance were observed in the nicarbazin (DNC) assay. The assay sensitivity was found to be reduced 40-fold in egg extract but only 4-fold in the feed extract. These reductions in sensitivity were believed to be due to interfering compounds present in the sample extract as the coccidiostats were added after the extraction to generate the calibration curves. All assays showed a loss in sensitivity when the calibration curves were constructed in egg or feed extracts, as opposed to buffer. It is possible that coccidiostats bind to co-extracted compounds that make them inaccessible for the antibody binding [37, 38].

Another phenomenon observed was a high background in feed for the lasalocid assay. Attempts to reduce this background such as filtration, did not succeed. This background is consistent, as it was continuously measured over 7 days. A significant part of this background was caused by the binding of the other antisera to the lasalocid-OVA-coated beads because the background was lower in the singleplex assay. Due to these effects, calibration curves have to be made in matrix-matched extracts of egg or feed for the correct calculation of sample concentrations.

For eggs, the limits of detection (LODs; mean, 3× standard deviation) of the assay were below the MRLs for narasin, diclazuril, nicarbazin and monensin, which was the desired situation. Even after the reduced sensitivity of the nicarbazin (DNC) assay in egg extract, it was still possible to use this assay because of the relatively high MRL. However, for the lasalocid assay the sensitivity was too high which may lead in practice to a higher number of false positive samples (Table 4).

**Table 4:** Comparison of assay sensitivities with the maximum (residue) levels

	Buffer (μg/L)		Egg (μg/kg)			Feed (μg/kg)		
	LOD	IC_50_	LOD	IC_50_	ML	LOD	IC_50_	ML
Narasin	0.01	0.3	0.01	0.2	2	0.1	2.7	700
Lasalocid	0.03	0.5	0.1	1.8	150	0.2	3	1,250
Diclazuril	0.2	1.9	0.5	6.3	2	0.3	5.3	10
Nicarbazin (DNC)	0.5	9	53	345	100	9	35	500
Monensin	0.01	0.3	0.1	1.2	2	1.5	7.5	1,250

The calibration curves in feed extract showed the diclazuril assay as the only assay with an IC_50_ close to the ML. The other four assays had too high sensitivities compared with the levels set within the EU (Table 4). In a singleplex assay, an extra dilution can overcome this problem. However, in this five-plex, the only option is to analyse the samples at two different dilutions because the diclazuril assay was not sensitive enough to allow an extra dilution step.

**Fig. 4:**
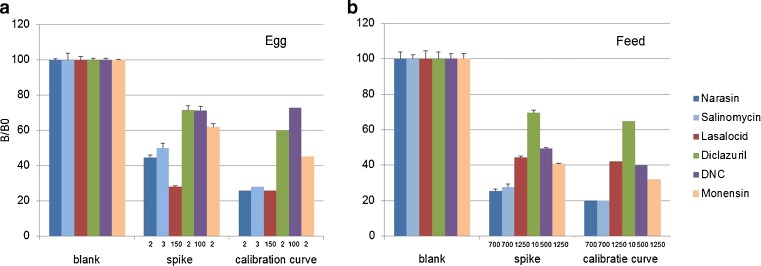
Average normalised results (*n* = 4) of blank and spiked egg (*left*) and feed samples (*right*) at the different MRLs and MLs and in comparison with the results obtained with the corresponding standard solution

The optimum extraction method was tested with a blank feed and egg sample and a blank feed and egg sample spiked with the different coccidiostats at their MLs or MRLs, respectively. With all spiked samples, significant inhibitions were observed which proved the application at the relevant levels (Fig. 4).

The intra assay variability was determined by measuring one egg or feed sample three times during 3 days and for the inter assay variability the same samples were measured ten times on the same day (Table 5). The assay precision was good because all variation coefficients were below 5 %.

**Table 5:** Precision of the screening method for the different analytes

	Egg		Feed	
	Intra-assay CV%	Inter-assay CV%	Intra-assay CV%	Inter-assay CV%
Narasin	1.4	2.3	1.7	0.3
Lasalocid	3.0	3.3	2.5	3.4
Diclazuril	2.3	3.0	1.7	1.3
Nicarbazin (DNC)	1.4	2.4	2.2	2.2
Monensin	1.8	3.4	4.4	2.8

The assay was tested for its feasibilities in real life. Preliminary cut-off levels were needed for this qualitative screening assay and they have been set at 0.5 M(R)L. Three incurred egg samples which were previously analysed with LC-MS [14, 39] were assayed with this FCIA. All samples had concentrations of 0.5 MRL. Two incurred egg samples contained only one coccidiostat: lacalocid or nicarbazin. The other incurred egg sample contained three coccidiostats: salinomycin, monensin and diclazuril. The results obtained with the three incurred samples in the screening assay were in accordance with the results obtained with the confirmatory method (Table 6). A fourth sample, containing unknown levels of narasin and nicarbazin was measured with both methods and in the screening higher inhibitions were seen than expected. The confirmatory method confirmed the higher level in this unknown egg sample.

**Table 6:** Comparison of the results obtained with the confirmatory and screening methods

Incurred or contaminated sample	Confirmatory method	Screening method^a^				
	MS (μg/kg)	Narasin	Lasalocid	Diclazuril	Nicarbazin (DNC)	Monensin
Egg 1	Lasalocid (40)	√	Non-compliant	√	√	√
Egg 2	Nicarbazin (DNC) (60)	√	√	√	Non-compliant	√
Egg 3	Salinomycin (1.5)	Non-compliant	√	Non-compliant	√	Non-compliant
	Monensin (1)					
	Diclazuril (1)					
Egg 4	Narasin (40)	Non-compliant	√	√	Non-compliant	√
	Nicarbazin (DNC) (80)					
Feed 1	Narasin (580)	Non-compliant	√	√	√	√
Feed 2	Salinomycin (910)	Non-compliant	√	√	√	√
Feed 3	Diclazuril (7)	√	√	Non-compliant	√	√
Feed 4	Nicarbazin (DNC) (570)	Non-compliant	√	√	Non-compliant	√
Feed 5	Monensin (1,000)	√	√	√	√	Non-compliant
Feed 6	Lasalocid (1,290)	√	Non-compliant	√	√	√

Non-compliant = above 0.5 MRL

^a^√ = compliant

Subsequently six feed samples contaminated with coccidiostat additives were analysed. Each feed sample contained one single coccidiostat at its ML. The results were in accordance with the spiked feed samples except for the cross-contaminated nicarbazin feed. This sample caused an inhibition in the nicarbazin assay but also unexpected inhibition in the narasin assay. It later transpired that for the preparation of the nicarbazin feed the premix Maxiban was used. This premix contains not only nicarbazin but also narasin and therefore the assay result was correct. No false positive nor false negative results were obtained with these real egg and feed samples.

The ability to store the extracts was tested. It was found that egg extracts which have been stored at 4 °C for 2 weeks could be measured without any problems. Feed extracts were stored in the same way but the standard deviation between the two measurements increased. The measurements were found to improve if the feed extracts were shaken for 10 min, followed by a centrifugation step at 2,400×*g* for 10 min before they were analysed. It could be concluded that this method gives the opportunity to do the whole procedure over a single day or to do the extraction on 1 day and analysis of the samples the following day, which could constitute an advantage for some routine control laboratories.

**Fig. 5:**
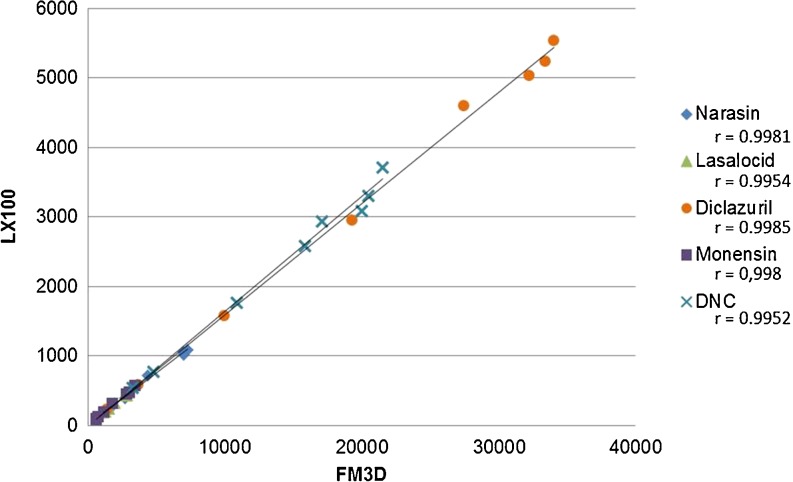
Comparison of the responses (MFIs) obtained with the Luminex LX100 and the FLEXMAP 3D® system with different calibration curves of the five-plex

During the multiplex assay development the new Luminex FM3D was introduced and to ensure that the assay could be transferred between the different platforms, compatibility with both instruments was investigated. With the five-plex coccidiostat assay, calibration curves were measured with both instruments (Fig. 5) and a very good correlation was observed with the FM3D showing 6 times higher responses (*r* ranging from 0.995 to 0.999 for the different assays). A complete validation for the application in eggs and feed will be performed taking into account internationally recognised standards such as for example the Commission Decision 2002/657/EC [40], the guidelines for validation of a screening methods [41] as well as other relevant concepts.

## Conclusions

In the present study, a bead-based FCIA screening was developed for the simultaneous detection of six priority coccidiostats: narasin, salinomycin, lasalocid, diclazuril, nicarbazin (DNC) and monensin in egg and feed. The presented bead-based assay format is very flexible and gives the opportunity to add other assays at any time, for example to extend the number of detectable coccidiostats.

Different conjugates were prepared and immobilised on the beads. These conjugates had a large influences on the sensitivities and the maximum signals of the assays and explained the difference in performance between the ELISA and the FCIA. One assay had the ability to detect two structurally related coccidiostats (salinomycin and narasin). When the singleplex assays were combined, the non-specific binding of antisera with non-associated beads was too high but could be reduced to acceptable levels by purifying two antisera. The direct coupling of the coccidiostats on the beads did not have the desired sensitivity and maximum binding.

We succeeded to develop a common and simple extraction procedure for egg and feed samples before analysis by FCIA. The final LODs for narasin/salinomycin, lasalocid, diclazuril, nicarbazin (DNC) and monensin in eggs were 0.01, 0.1, 0.5, 53 and 0.1 μg/kg and in feed 0.1, 0.2, 0.3, 9 and 1.5 μg/kg, respectively. This demonstrated that a five-plex FCIA could detect 6 coccidiostats simultaneously in eggs and feed at their MRLs and MLs, respectively. Spiked and incurred egg and feed samples were analysed and no false positive or false-negative positive results were obtained. A complete validation will be performed. The assays were tested for their compatibility with the Luminex LX100 and FM3D and a very good correlation was observed between both flow cytometers demonstrating adequate transferability between instrumental platforms.
